# The rhizosphere microbiome and its influence on the accumulation of metabolites in *Bletilla striata* (Thunb.) Reichb. f

**DOI:** 10.1186/s12870-024-05134-0

**Published:** 2024-05-17

**Authors:** Shiqing Li, Xiaomei Li, Yueyu Ye, Man Chen, Haimin Chen, Dongfeng Yang, Meiya Li, Fusheng Jiang, Xiaobo Zhang, Chunchun Zhang

**Affiliations:** 1 College of Pharmaceutical Sciences, Zhejiang Chinese Medical University, Hangzhou, 310053 Zhejiang China; 2 Key Laboratory of Plant Secondary Metabolism and Regulation of Zhejiang Province, College of Life Sciences and Medicine, Zhejiang Sci-Tech University, Hangzhou, 310018 Zhejiang China; 3 Academy of Chinese Medical Sciences, Zhejiang Chinese Medical University, Hangzhou, 310053 Zhejiang China; 4 College of Life Sciences, Zhejiang Chinese Medical University, Hangzhou, 310053 Zhejiang China

**Keywords:** *Bletilla striata*, Rhizosphere microbes, Metabolites

## Abstract

**Background:**

*Bletilla striata* (Thunb.) Reichb. f. (*B. striata*) is a perennial herbaceous plant in the Orchidaceae family known for its diverse pharmacological activities, such as promoting wound healing, hemostasis, anti-inflammatory effects, antioxidant properties, and immune regulation. Nevertheless, the microbe-plant-metabolite regulation patterns for *B. striata* remain largely undetermined, especially in the field of rhizosphere microbes. To elucidate the interrelationships between soil physics and chemistry and rhizosphere microbes and metabolites, a comprehensive approach combining metagenome analysis and targeted metabolomics was employed to investigate the rhizosphere soil and tubers from four provinces and eight production areas in China.

**Results:**

Our study reveals that the core rhizosphere microbiome of *B. striata* is predominantly comprised of *Paraburkholderia*, *Methylibium*, *Bradyrhizobium*, *Chitinophaga*, and *Mycobacterium*. These microbial species are recognized as potentially beneficial for plants health. Comprehensive analysis revealed a significant association between the accumulation of metabolites, such as militarine and polysaccharides in *B. striata* and the composition of rhizosphere microbes at the genus level. Furthermore, we found that the soil environment indirectly influenced the metabolite profile of *B. striata* by affecting the composition of rhizosphere microbes. Notably, our research identifies soil organic carbon as a primary driving factor influencing metabolite accumulation in *B. striata*.

**Conclusion:**

Our fndings contribute to an enhanced understanding of the comprehensive regulatory mechanism involving microbe-plant-metabolite interactions. This research provides a theoretical basis for the cultivation of high-quality traditional Chinese medicine *B. striata.*

**Supplementary Information:**

The online version contains supplementary material available at 10.1186/s12870-024-05134-0.

## Background

The concept of rhizosphere soil was first proposed by German scientist Hiltner in 1904 to describe the soil influenced by plant roots, which subsequently prompted research into rhizosphere microbes. These microbes are attracted by plant root exudates [1–3] and are abundant in rhizosphere soil, exerting a significant impact on various aspects of plant biology, including nutrition [4, 5], growth [6, 7], disease resistance, and stress tolerance [8–10]. In recent years, an increasing number of studies have shown that biological factors such as endophytes and rhizosphere microbes directly or indirectly affect the growth, traits, metabolism, and other attributes of medicinal plants. Consequently, this leads to variations in the quality of medicinal plants across different regions and environments. For example, Su et al. [11] observed an enrichment of terpenoid backbone biosynthetic genes in the rhizosphere of *Citrus reticulata* ‘Chachi’ within its core area compareed to non-core areas. Furthermore, they demonstrated that inoculation with Strep-4, a strain of isolated *Streptomyces* abundant in core rhizosphere soil, significantly increased the concentration of monoterpenes in *Citrus reticulata* ‘Chachi’. Additionally, Zhong et al. [12] through a comprehensive analysis involving microbe-plant-metabolites inferred that *Lysobacter* and *Rhodoplanes* in the rhizosphere of *Glycyrrhiza uralensis Fisch* could affect the accumulation of liquiritin and glycyrrhizic acid.

*B. Striata,* a perennial herbaceous plant belonging to the Orchidaceae family, is primarily distributed in southern Shaanxi, southeastern Gansu, Jiangsu, Anhui, Zhejiang, and other regions of China. Modern research has highlighted the significant role of *B. striata* in wound healing [13–16], hemostasis [17], anti-inflammatory [18], antioxidant [19, 20], whitening [21, 22], and immune regulatory activity [23]. Current researches on *B. striata* components are mainly focused on isolating and identifying chemical compounds [24, 25], quality control [26], and pharmacological activities [27, 28]. Research into factors influencing the quality of *B. striata* mainly focuses on varieties, cultivation and planting techniques, processing methods, and other aspects [29–31]. In terms of microecology, most studies have mainly focused on endophytic fungi [32] and mycorrhizal fungi [33–35]. However, there have been no reports regarding the composition, diversity, and function of rhizosphere microbes in *B. striata*, and there are limited studies on the correlation between *B. striata* quality and biological factors, especially in the field of rhizosphere microbes.

Samples of fresh *B. striata* tubers, bulk soil, and rhizosphere soil were collected from eight production areas spanning four provinces in China. A comprehensive approach involving targeted metabolomics, amplicon sequencing, and metagenomic sequencing techniques, combined with structural equation modeling (SEM) was employed to explore the interrelationships between soil physics and chemistry and rhizosphere microbes and metabolites. This study aims to provide a theoretical basis for the cultivation of high-quality traditional Chinese medicine *B. striata*.

## Results

### Taxonomic characteristics of rhizosphere microbes in *B. striata*

**Fig. 1:**
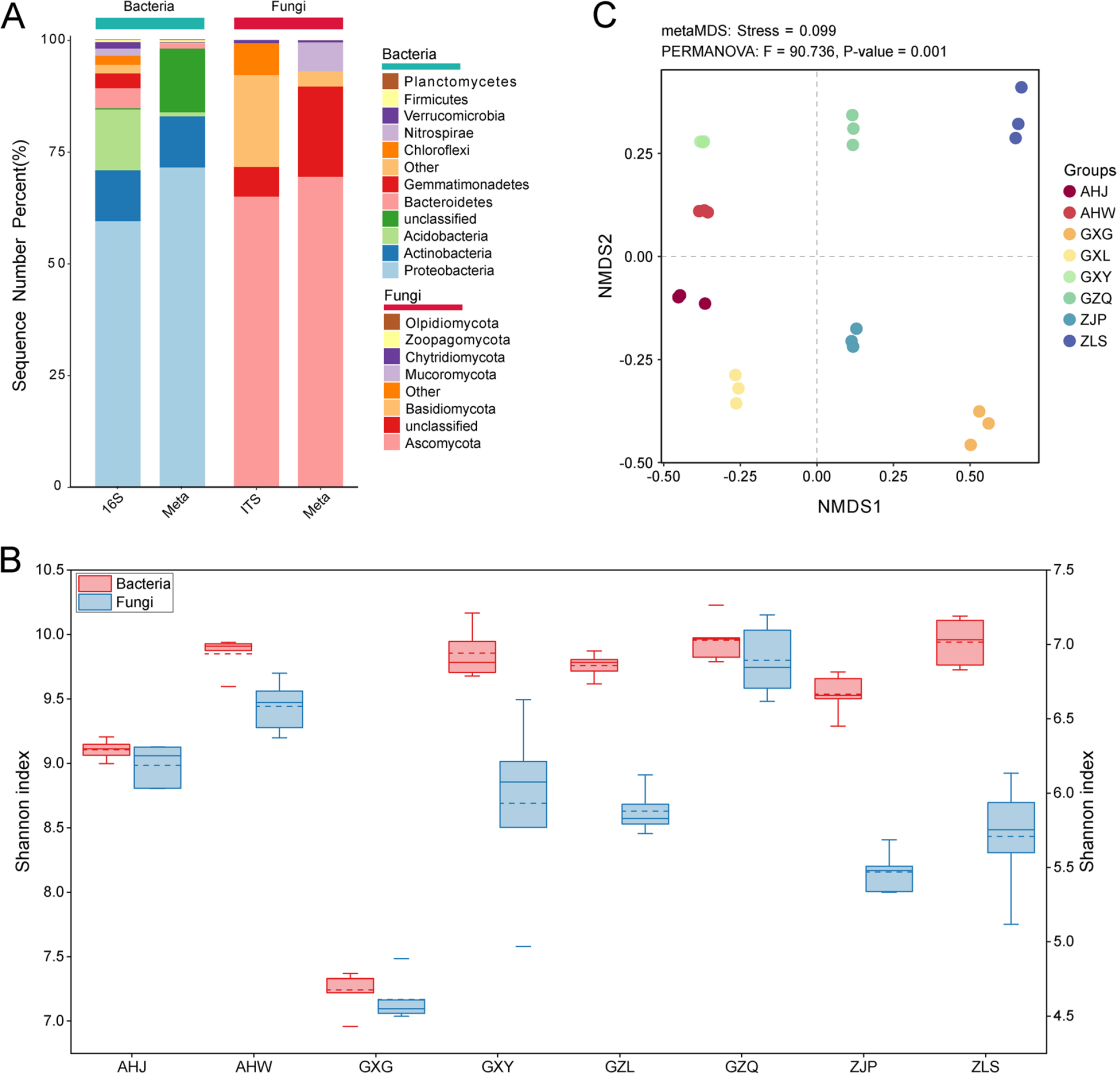
Comparison of the composition and diversity of rhizosphere microbial communities in *B. striata* from different areas. **A** Rhizosphere microbial composition at the phylum level, based on amplicon sequence and metagenomic data. **B** Metagenomic data for non-metric multidimensional scaling (NMDS) analysis based on the Bray Curtis distance. **C** Shannon index of the alpha diversity of rhizosphere microbes of *B. striata* in different habitat regions, based on the amplicon data (solid line represents the median Shannon index, and dashed line represents the mean Shannon index)

The taxonomic composition of the rhizosphere microbes of *B. striata* was determined through metagenomic sequencing, supplemented by amplicon sequences, to obtain taxonomic annotation results: prokaryotes (bacteria and archaea) accounted for 99.01% of the total annotations, eukaryotes accounted for 0.96% of the total annotations, and virus genes only accounted for 0.02% of the total annotations. The main bacterial phyla found in the rhizosphere soil of *B. striata* included Proteobacteria, Actinobacteria, Acidobacteria, and Bacteroidetes, while the fungal phyla were Ascomycota, Basidiomycota, and Mucor mycota (Fig. 1A).

Utilizing three non-parametric indices (Chao1, Shannon, and Simpson indices) to assess the rhizosphere soil samples, significant differences in alpha diversity of rhizosphere microbes from different origins were observed (Kruskal Wallis test, bacteria, H = 30, *p* < 0.01; fungi, H = 33, *p* < 0.01). Notably, the diversity of bacterial and fungal communities in the rhizosphere soil of Guilin, Guangxi was significantly lower than that of other production areas (Fig. 1B). NMDS analysis based on Bray Curtis distance revealed significant differences in the composition of rhizosphere microbial communities of *B. striata* among different habitats (Fig. 1C). Furthermore, we compared the relative abundance of rhizosphere microbes in different habitats between high (phyla) and low (genus) classification levels through metagenomic sequences. We identified multiple bacterial phyla with higher relative abundance in the *B. striata* rhizosphere in Jinzhai, Anhui, and Guilin, Guangxi such as Proteobacteria, while Actinobacteria had a higher relative abundance in Lishui, Zhejiang (Supplementary Fig. 1A). Various bacterial genera including *Sphingobium* and *Burkholderia* exhibited significant differences in enrichment within the *B. striata* rhizosphere across diverse habitats (Supplementary Fig. 1B). Moreover, there are unique and differential species of rhizosphere microbes present within *B. striata* from different origins. Microbes with an LDA score > 4 were selected as differential species revealing all unique and differential microbes concentrated within the bacterial domain. Notably *B. striata* from Lishui, Zhejiang, displayed the most significant differential microbial species reaching up to 15 species within its respective rhizospheric environment (Supplementary Fig. 2).

**Fig. 2:**
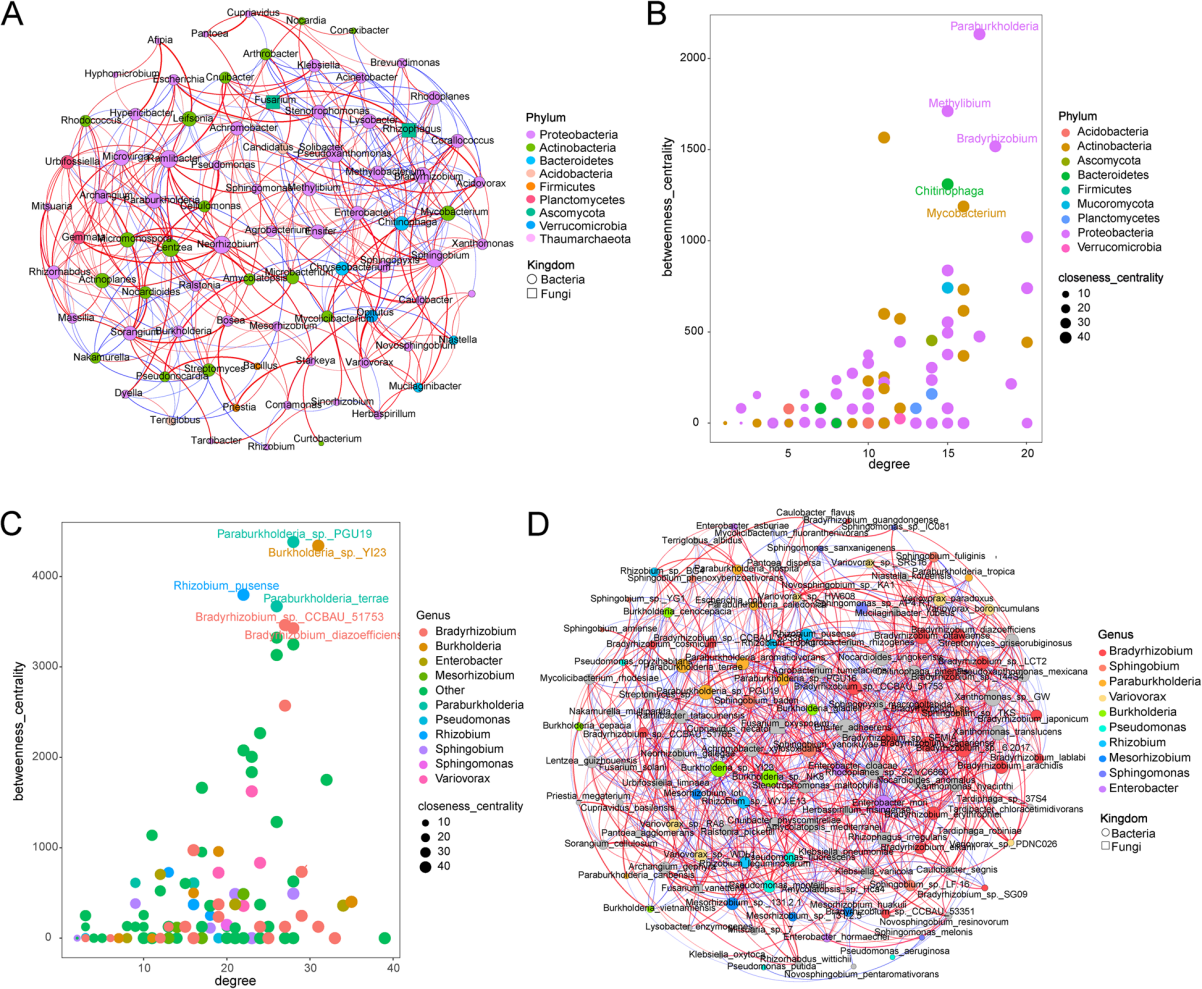
Analysis of the characteristics of the rhizosphere core microbes of *B. striata*. **A** Co-occurrence network analysis diagram of the rhizosphere microbes of *B. striata* at the genus level. Nodes represent microbial genera, node size represents the degree, and the thickness of the line between nodes indicates the size of the correlation coefficient between them. The edge represents the Spearman’s correlation coefficient (*R* > 0.6, *p* < 0.05). **B** Core microbial groups at the genus level with significant centrality (a measure of the importance of nodes). **C** Core microbial groups at the species level. **D** Analysis diagram of the co-occurrence network of the rhizosphere microbes at the species level

### Core taxa of rhizosphere microbial community in *B. striata*

Given the metagenomic sequence’s ability to offer comprehensive taxonomic information and consistent community composition results with amplicon sequencing, it was chosen for identifying the core rhizosphere microbial communities of *B. striata*. Following the methodology outlined by Dong [36] and Xu J [37], the core group standard of rhizosphere was determined by taking into account all the habitats of *B. striata*; microbial communities or species existed in over 75% of *B. striata* samples, and their relative abundance was greater than 0.01%. Additionally, shared network analysis was conducted using Spearman’s correlation coefficient (*p*), where an absolute value greater than 0.6 was utilized as a threshold for inclusion.

Based on genus-level analysis, the core taxonomic group of the microbial community formed a rhizosphere microbial network consisting of 83 nodes and 448 edges (Fig. 2A). The average path length between all node pairs was 2.5, with an average degree of 10.8, a clustering coefficient of 0.50, and a modularity index of 0.43. According to network connectivity statistics (degree, closeness centrality, and betweenness centrality), *Paraburkholderia*, *Methylibium*, *Bradyrhizobium*, *Chitinophaga*, and *Mycobacterium* were defined as the core rhizosphere microbial genera (Fig. 2B), all belonging to the phylum Proteobacteria.

Based on species-level analysis, the core taxonomic group of the microbial community was obtained, and the rhizosphere microbial network of *B. striata* included 128 nodes and 1089 edges (Fig. 2D). The average path length between all node pairs was 2.4 edges, with an average degree of 17, a clustering coefficient of 0.51, and a modularity index of 0.41. In accordance with the network connectivity statistics, *Paraburkholderia_sp._PGU19*, *Paraburkholderia_terrae*, *Burkholderia_sp._YI23*, *Rhizobium_pusense*, and *Bradyrhizobium_sp. CCBAU_51753, Bradyrhizobium_diazoefficiens* and *Rhizobium_pusense*, were identified as core rhizosphere microbial species (Fig. 2C), with two species belonging to *Paraburkholderia* and two species belonging to *Bradyrhizobium*. These findings are consistent with the core rhizosphere microbial community at the genus level. Moreover, as depicted in Fig. 2D, there was a positive correlation among microbial species within the genus *Bradyrhizobium*, such as *Bradyrhizobium diazoefficiens*, *Bradyrhizobium_ottawaense*, *Bradyrhizobium_sp._144S4*, and *Bradyrhizobium_ sp._LCT2*, indicating that synergistic effects are more likely to occur when microbial species belong to the same genus.

### Functional characteristics analysis of rhizosphere microbes in the rhizosphere of *B. striata*

**Fig. 3:**
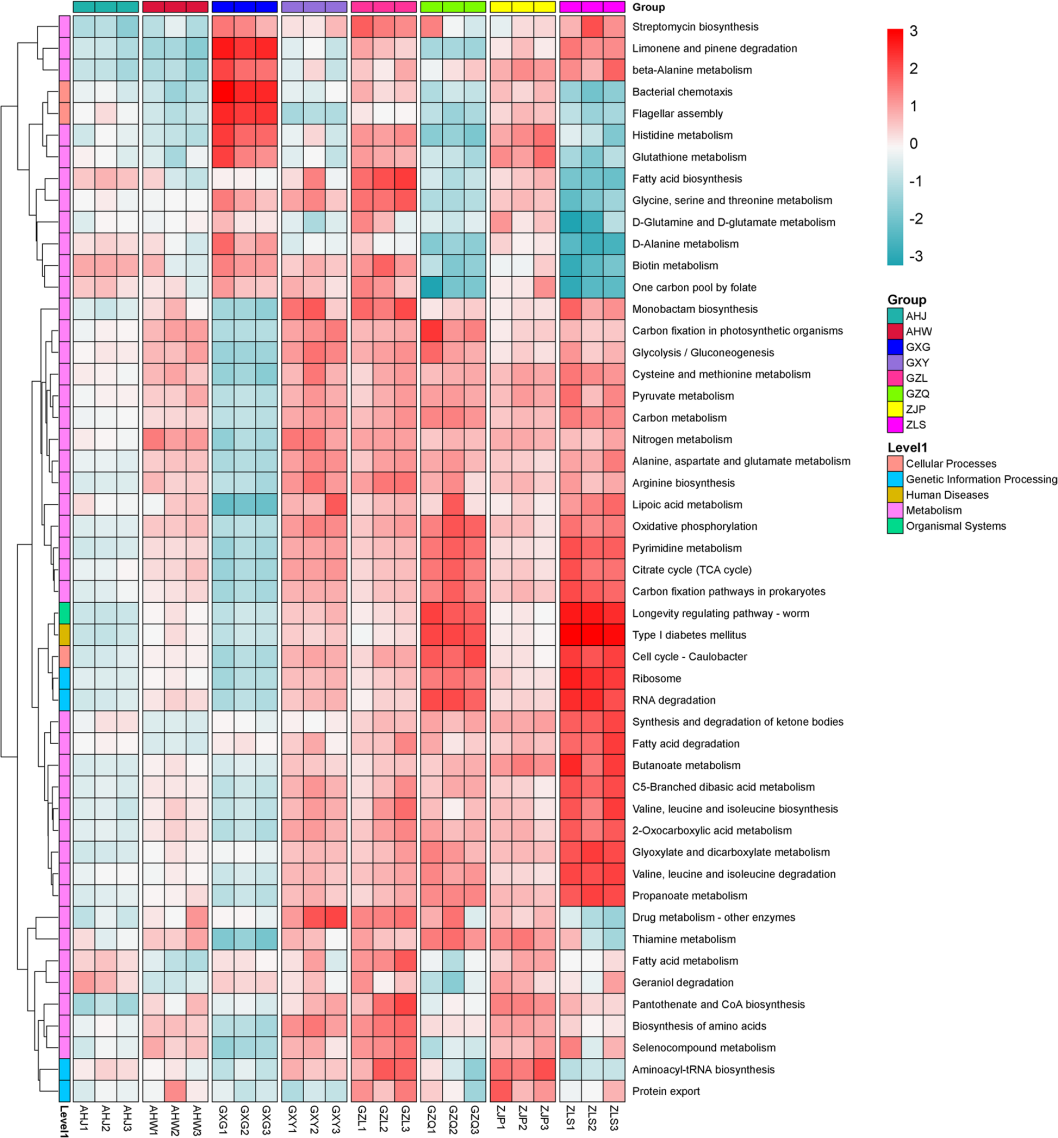
Heat map analysis of the top 50 functional pathways at Level 3

**Fig. 4:**
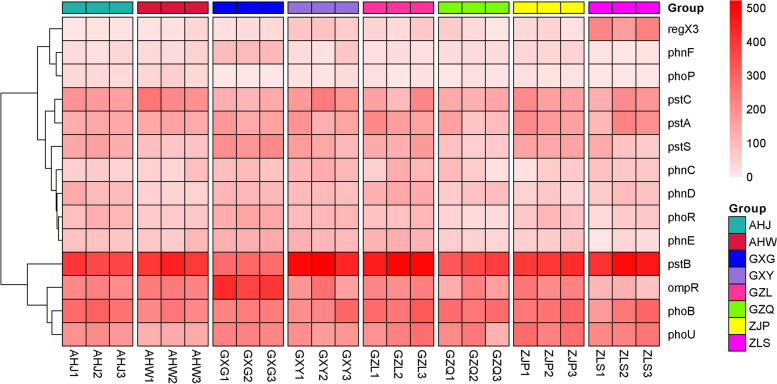
Core rhizosphere KO grouping and clustering heatmap

Through an analysis of the KEGG Orthology (KO) database, a total of 6153 KOs were identified from rhizosphere samples, and 47 annotated KEGG secondary pathways. Notably, the most prevalent metabolic pathways included amino acid metabolism, carbohydrate metabolism, and metabolism of cofactors and vitamins metabolism, accounting for 12.75%, 11.43%, and 8.19%, respectively. Referring to the method of Xu et al. [37], a rhizosphere enrichment threshold of 75% was utlized to define the core functional characteristics of the rhizosphere microbiome of *B. striata*. These core functional features primarily encompass microbial interactions with both host plants, and other microbes, as well as potential nutrient acquisition pathways in which rhizosphere microbes may be involved. According to the top 50 functional pathways at level 3 (Fig. 3), it was found that pathways related to plant–microbe and microbe-microbe interactions, such as flagella assembly, bacterial chemotaxis, and carbon fixation pathways in prokaryotes, are centrally expressed in the rhizosphere microbes of *B. striata*. Based on the analysis of KEGG level 3 functional pathways, we found that rhizosphere microbes of *B. striata* have rich amino acid synthesis and metabolism pathways (including alanine aspartate glutamate metabolism, histidine metabolism, glycine, serine, and threonine metabolism, cysteine and methionine metabolism, arginine biosynthesis, and valine, leucine, and isoleucine biosynthesis) (Fig. 3). In addition, many core rhizosphere KOs may also benefit plants by participating in multiple nutrient acquisition pathways, such as phosphate transport (pstA, pstB, pstC, pstS, phnC, phnD, phnE, phnF, phoU) and phosphate regulatory responses (phoB, phoP, phoR, ompR, regX3) (Fig. 4).

**Fig. 5:**
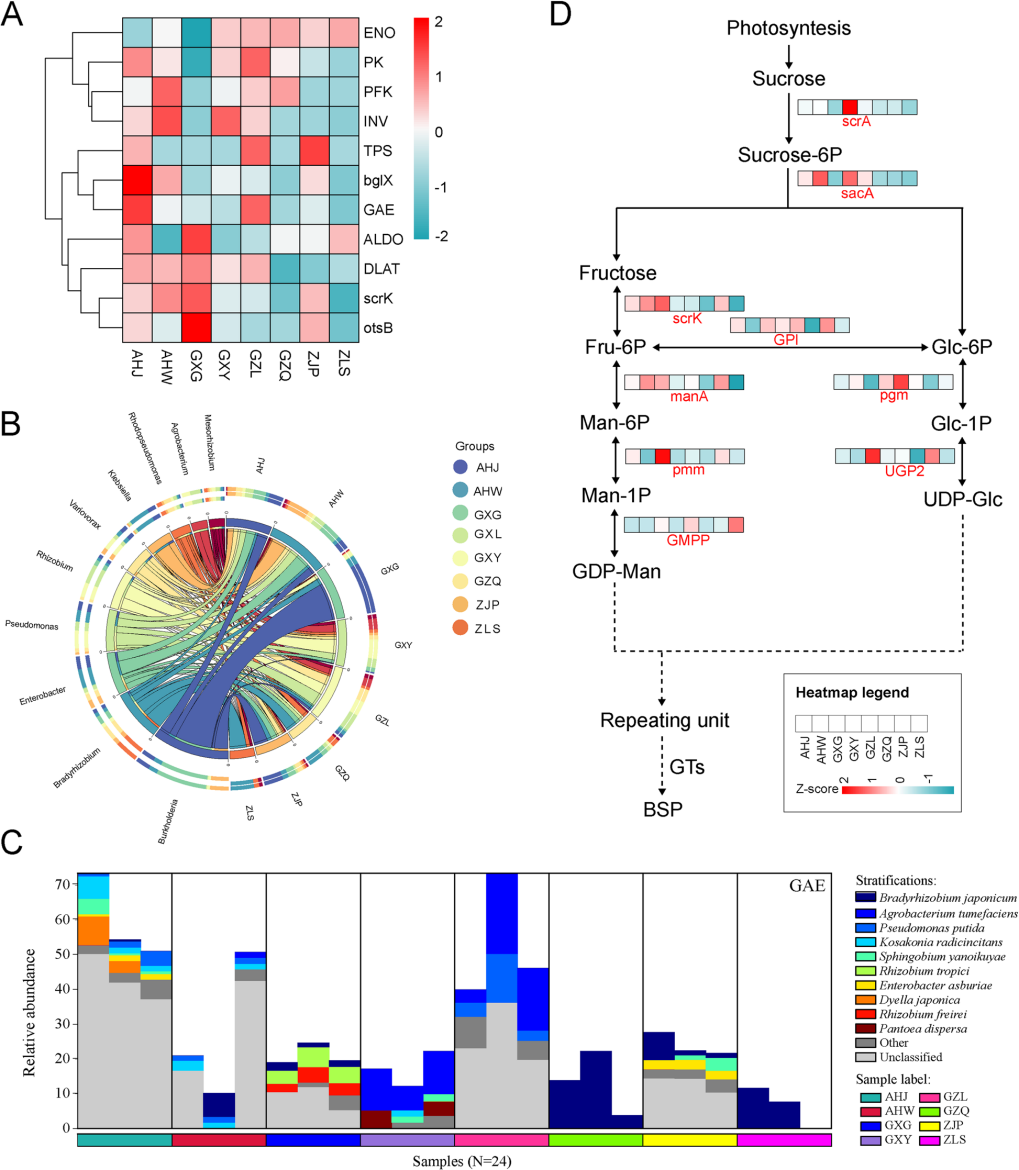
Analysis of the functional characteristics of the rhizosphere microbial community in *B. striata*. **A** After zero mean normalization, the abundance heat map analysis of 11 genes related to militarine biosynthesis. **B** Circos diagram of microbes that may be involved in militarine biosynthesis. **C** Species-stratified histogram of the GAE gene in militarine biosynthesis. **D** Biosynthetic pathway of BSP

Previous studies have highlighted the potential for microbial metabolic capacity and related genes to contribute to the synthesis of medicinally active ingredients in medicinal plants [38]. For instance, preliminary screenings by Liu et al. [39] suggested that specific genes such as bgIX (β-d-Glucosidases), otsB (trehalose-phosphate phosphatase), TPS (trehalose-6-phosphate synthase), and GAE (UDP-glucuronate 4-epimerase) may play pivotal roles in militarine synthesis. Additionally, Niu et al. [40] provided molecular-level insights into the pathway of BSP (*B. striata* polysaccharide) synthesis and metabolism. Therefore, the current investigation focuses on identifying key genes involved in the biosynthesis pathways of militarine and BSP within the rhizosphere microbes of *B. striata.* A comprehensive analysis conducted across various production areas revealed a richness of genes related to militarine synthesis in the rhizosphere microbiota of Anhui Jinzhai, including bgIX and GAE. In contrast, Liupanshui in Guizhou exhibited an abundance of genes associated with militarine synthesis, such as GAE and TPS. Research has indicated that bgIX encodes β-D-glucosidase, a crucial enzyme in cellulose degradation, which possesses both glycoside hydrolysis and glycosyltransferase activities [41]. The GAE gene encodes UDP-glucuronate 4-epimerase, which plays an important regulatory role in sugar transport, mainly catalyzing the mutual transformation of UDP-glucuronic acid and UDP-D-Galacturonic acid [42]. The TPS gene is capable of catalyzing the synthesis of trehalose 6-phosphate from UDP-glucose and glucose-6-phosphate, ultimately leading to enhanced trehalose accumulation and improved plant stress resistance. Furthermore, it has been observed that overexpression of TPS also modulates the expression of genes associated with plant abscisic acid, glucose, and anthocyanin synthesis pathways [43]. Militarine is a glycoside compound found in abundance in *B. striata*, and it is formed through the dehydration of the hydroxyl amino thiol group of monosaccharides or oligosaccharides and the hemiacetal hydroxyl group of another molecule. Based on metabolome and transcriptome analysis of *B. striata* suspension culture cells, as well as fluorescence quantitative PCR validation, Liu et al. [39] proposed that bgIX, TPS, and GAE genes are closely associated with the biological synthesis and accumulation of militarine. Therefore, it is suggested that the rhizosphere microbes of *B. striata* from these two regions may possess significant potential for promoting militarine biosynthesis (Fig. 5A and B). The GAE genes enriched in both regions were annotated into 15 genera and 21 species across all regions, with a predominant presence of microbial species such as Bradyrhizobium japonicum, Agrobacterium tumefaciens, Pseudomonas putida, and Rhizobium tropici. Notably, four of these species belonged to the genus Rhizobium (Fig. 5C). The heat map analysis revealed that the abundance of most of the 21 species annotated by GAE was significantly higher in Jinzhai and Anhui compared to other production areas (Supplementary Fig. 3), aligning with the high expression levels of GAE genes. This consistency indicates a high level of data reliability. Furthermore, our analysis identified that pivotal genes associated with the biosynthesis of BSP within rhizosphere microbiota from diverse habitats, such as pmm, UGP2, GPI, manA, and scrK, demonstrated heightened expression levels in Guilin, Guangxi and Pan'an, Zhejiang (Fig. 5D).

### Analysis of the correlation between soil factors and rhizosphere microbes of *B. striata*

**Fig. 6:**
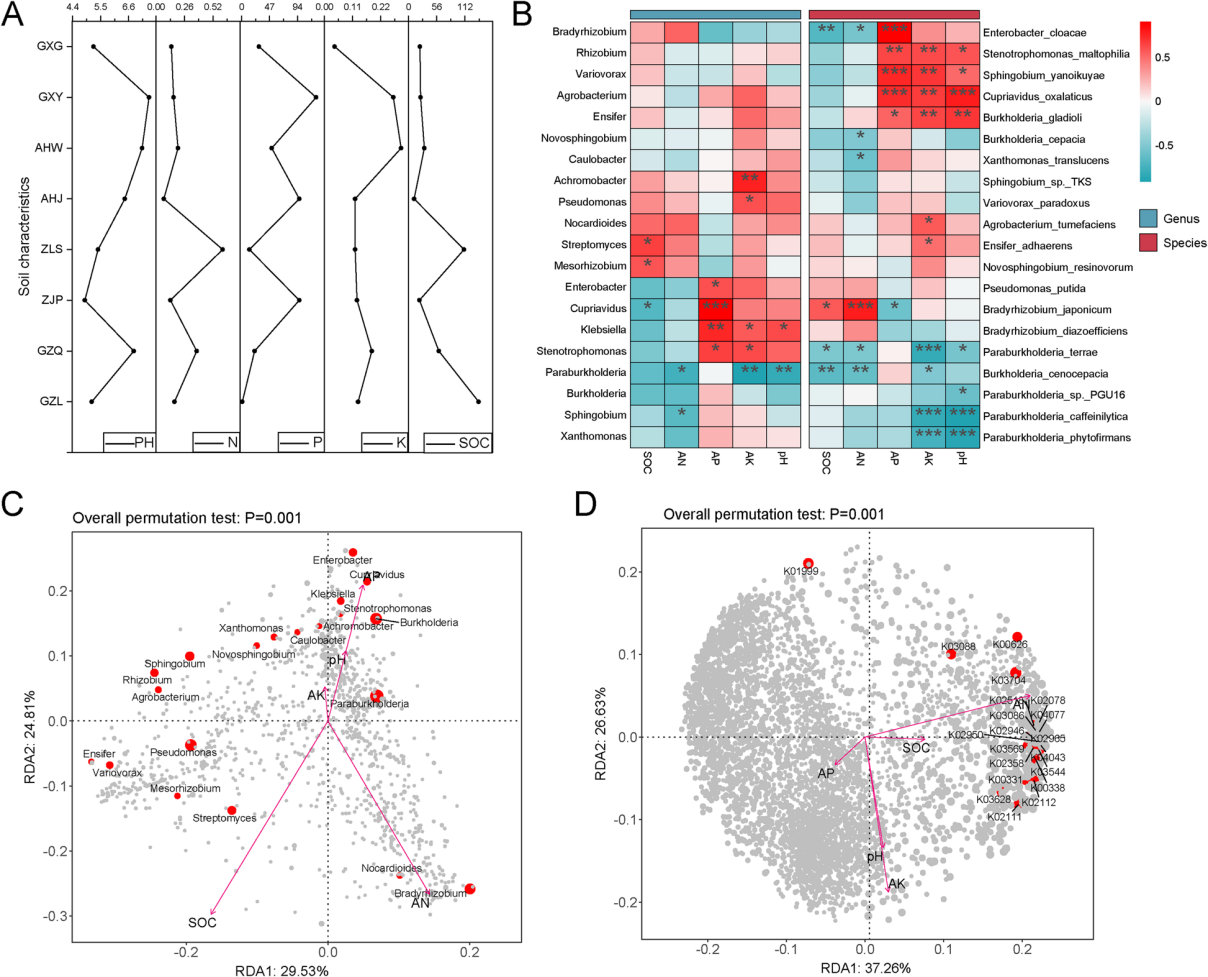
Correlation analysis between rhizosphere microbes and soil physicochemical factors in *B. striata*. **A** Inverted diagram of the soil physical and chemical properties from different production areas. **B** Heat map analysis of the correlation between the composition of rhizosphere microbial communities and the soil physical and chemical properties. **C** RDA of microbial community composition and soil physicochemical properties in the rhizosphere of *B. striata.* **D** RDA of the top 20 KOs in the rhizosphere microbes of *B. striata* and soil physicochemical properties

The results of the soil physical and chemical properties from the eight production areas are shown in Fig. 6A. It was observed that the soil pH varied significantly among different production areas, generally exhibiting weak acidity. The highest pH value was found in the soil from the Guangxi Medical Botanical Garden production area. Additionally, a notable finding was the significantly higher soil organic carbon content in Liupanshui, Guizhou, compared to other producing areas (*p* < 0.05). Previous research has indicated that soil physicochemical properties not only influence soil fertility but also play a role in shaping the structure and diversity of rhizosphere microbiota [44]. The correlation heat map showed a significant negative correlation (*p* < 0.01) between Paraburkholderia and soil-available potassium and soil pH, consistent with the correlations involving four microbial species belonging to Paraburkholderia (Fig. 6B). Redundancy analysis (RDA) revealed that various soil physical and chemical factors affect the composition of the rhizosphere microbial community of *B. striata*, with soil-available nitrogen and soil organic carbon being the main factors affecting the composition of the rhizosphere microbial community (Fig. 6C). Upon analyzing the top 20 KOs with the highest degree of correlation between rhizosphere microbes and soil physicochemical factors, we observed a strong positive correlation between soil-available nitrogen and most of these KOs, indicating a significant association between soil-available nitrogen levels and the majority of the identified KOs (Fig. 6D).

### Analysis of factors influencing the metabolic accumulation of *B. striata* tubers.

**Fig. 7:**
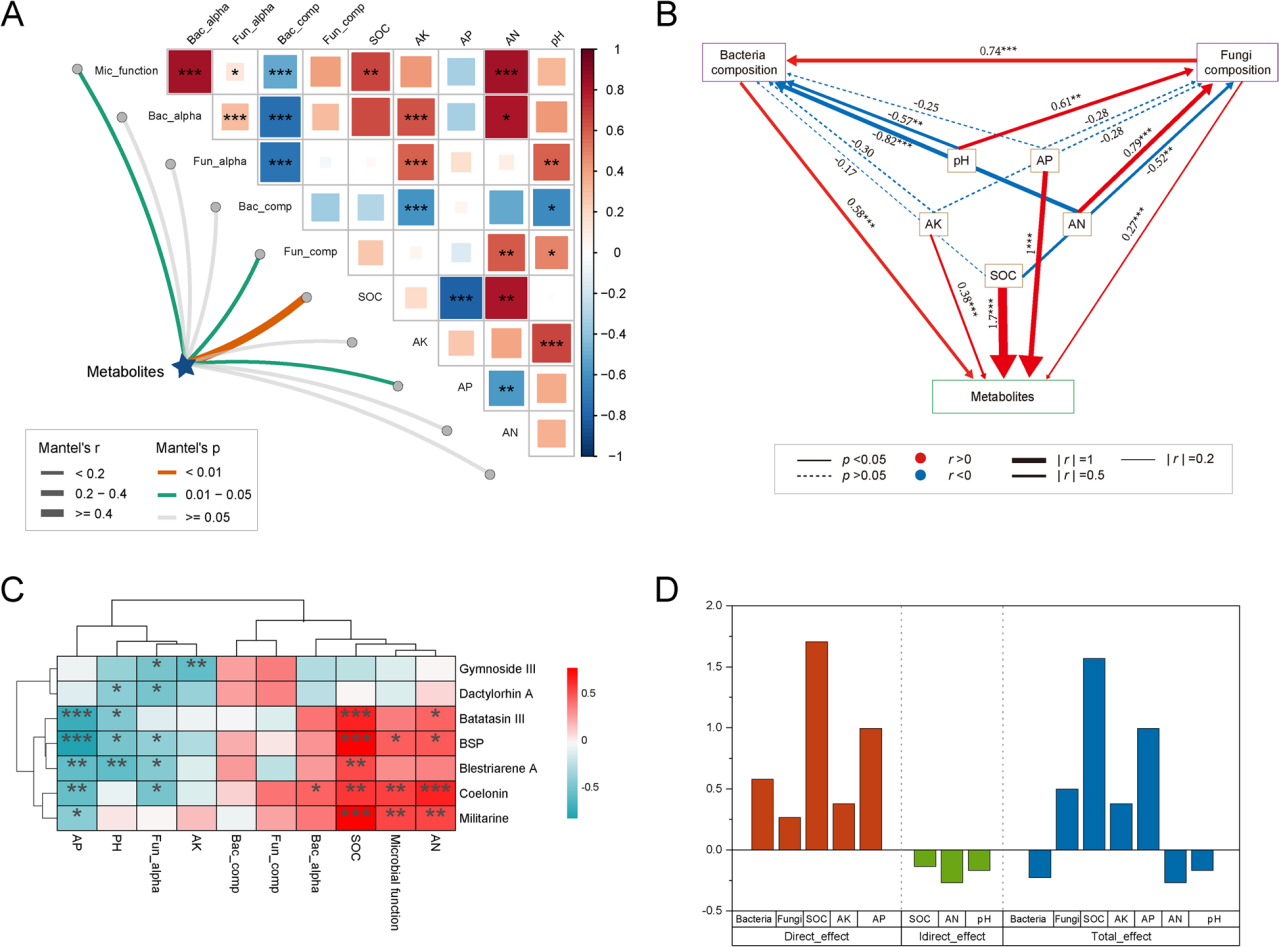
Analysis of the factors affecting metabolite accumulation in *B. striata*. **A** Correlation heat map of environmental factor indicators. Spearman's correlation coefficient is represented by the color gradient. Using the Mantel test, a correlation analysis was conducted between the metabolites of *B. striata* and each influencing factor. The edge width corresponds to Mantel's r statistic of the distance correlation, and the edge color represents statistical significance. **B** Structural equation modeling: Red represents positive correlation, blue represents negative correlation, and the words on the line represent path coefficients; *** *p* < 0.001, ** *p* < 0.01, and *p < 0.05. Model fitness indicated that the model was good: χ 2 / df = 0.88, *P* = 0.415, GFI = 1, SRMR = 0.003. **C** Heat map showing the correlations between environmental factors and various metabolites. **D** Bar chart showing the standardized effects (direct, indirect, and total) based on the structural equation model

Using HPLC and sulfuric acid-phenol methods, the metabolite content in *B. striata* tubers from various regions was analyzed. The findings revealed significantly higher levels of the indicator component, militarine, in *B. striata* tubers from Liupanshui, Guizhou and Lishui, Zhejiang compared to other regions. And the content of BSP was found to be consistent with that of militarine (Table 1). A correlation analysis was conducted examine the relationship between metabolic components and environmental factors, including the microbiome and soil physicochemical properties. The results showed a significant positive correlation between available soil nitrogen, soil organic carbon, rhizosphere microbial diversity, and rhizosphere microbial function. There was a significant positive correlation between metabolites of *B. striata* and soil organic carbon, especially the metabolites of militarine, BSP, batatasin III, blestriarene A, and coelonin (Fig. 7A and C). Furthermore, we employed structural equation modeling to investigate the influence of biological and abiotic factors on metabolite accumulation in *B. striata*. The results demonstrated that bacterial composition (path coefficient = 0.58, *p* < 0.001), fungal composition (path coefficient = 0.27, *p* < 0.001), soil organic carbon (path coefficient = 1.7, *p* < 0.001), soil-available phosphorus (path coefficient = 1,* p* < 0.001), and soil-available potassium (path coefficient = 0.38, *p* < 0.001) had direct and significantly effects on metabolite accumulation in *B. striata*. Soil organic carbon, alkaline nitrogen, and pH were identified as indirect influencers of *B. striata* metabolite accumulation through their impact on the composition of bacteria and fungi (Fig. 7B). After standardizing the effect values, we found that soil organic carbon had the highest direct and total effects (Fig. 7D), indicating that soil organic carbon was the most important driving factor affecting metabolite accumulation in *B. striata*. In addition, the structural model also indicated that fungal community composition had a certain positive regulatory effect on bacterial community composition (path coefficient = 0.74, *p* < 0.001). However, compared to the composition of fungal communities, the composition of bacterial communities played a more important role in metabolite accumulation in *B. striata*. In summary, considering the intricate interactions among soil, plants, and rhizosphere microbial communities, both abiotic and biological factors have distinct effects on metabolite accumulation in *B. striata*.

**Table 1:** Content of compounds in different regions (*n* = 3)

Sampling location	Compound content (mg/g)						
	Dactylorhin A	Gymnoside III	Militarine	Coelonin	Batatasin III	Blestriarene A	BSP
AHJ	6.009 ± 0.9535 cd	5.2388 ± 0.3409 c	38.1998 ± 0.9125 c	0.0408 ± 0.0008 c	0.0187 ± 0.0013 b	0.018 ± 0.0005 c	174.0958 ± 36.4156 d
AHW	7.1332 ± 0.3567 c	4.0848 ± 0.148 c	49.8225 ± 1.4086 c	0.0892 ± 0.0173 c	0.0318 ± 0.002 b	0.0298 ± 0.0057 bc	205.2137 ± 9.2506 cd
GXG	12.219 ± 2.0458 b	9.0032 ± 2.9097 b	47.4378 ± 18.6558 c	0.2958 ± 0.0536 bc	0.0225 ± 0.0071 b	0.04 ± 0.002 bc	244.8962 ± 15.6003 c
GXY	5.9167 ± 0.6386 cd	3.0998 ± 0.2694 c	115.1148 ± 16.0618 b	0.1935 ± 0.0275 bc	0.011 ± 0.0013 b	0.0283 ± 0.0015 bc	212.1596 ± 19.1088 cd
GZL	6.106 ± 0.2944 cd	2.9293 ± 0.1366 c	158.7448 ± 5.3211 a	0.2465 ± 0.1048 bc	0.087 ± 0.0065 a	0.1328 ± 0.0361 a	471.9021 ± 49.0625 a
GZQ	4.4028 ± 1.515 d	4.4413 ± 1.3803 c	47.4432 ± 7.7451 c	0.8005 ± 0.4339 a	0.0962 ± 0.0749 a	0.1265 ± 0.0782 a	325.272 ± 35.9659 b
ZJP	7.7395 ± 0.0398 c	8.639 ± 0.1804 b	45.3762 ± 0.5105 c	0.1743 ± 0.0071 bc	0.046 ± 0.003 ab	0.1112 ± 0.0078 a	242.2628 ± 12.4679 c
ZLS	15.8213 ± 0.4605 a	11.8132 ± 0.3018 a	124.511 ± 2.5963 b	0.413 ± 0.0212 b	0.054 ± 0.0052 ab	0.081 ± 0.0139 ab	370.5274 ± 38.3959 b
χ2	43.517	23.689	74.838	6.772	4.171	7.06	33.179
P	< 0.01	< 0.01	< 0.01	< 0.01	< 0.05	< 0.01	< 0.01

All data are presented as mean ± standard error according to Tukey’s test. Different lowercase letters in the same column indicate significant differences (*p* < 0.05)

## Discussion

The rhizosphere, an ecological niche facilitating interactions among plant, soil, and microorganisms, is a highly active zone for material and energy exchange [45]. Plants have the ability to influence the composition of rhizosphere microbiota through secretions and immune systems. Concurrently, rhizosphere microbes actively participate in processes, such as plant development, nutrient absorption, and stress response, through metabolic activities [46, 47]. Consequently, the rhizosphere microbiota serves as a critical factor influencing the intricate relationships between plants, soil, and microbes while playing a pivotal role in maintaining plant health. The results of this study showed that the dominant microbes in the rhizosphere of *B. striata* were mainly prokaryotes, which accounted for 99% of the total microbial community. Among these, Proteobacteria emerged as the dominant phylum, followed by Actinobacteria, Acidobacteria, and Bacteroidetes. Zuo et al. [48]found that Proteobacteria, Acidobacteria, Actinobacteria, and Bacteroidetes are the dominant bacterial groups in the rhizosphere of *Dendrobium officinale*. Similarly, high-throughput Illumina MiSeq sequencing of the rhizosphere soil of *Gymnadenia conopsea* identified Proteobacteria, Actinobacteria, Acidobacteria, and Bacteroidetes as the main groups [49]. Our findings corroborate these results, and it appears that Proteobacteria, Acidobacteria, Actinobacteria, and Bacteroidetes are the predominant bacterial communities in orchid plant rhizospheres. However, it is noteworthy that these microbiota communities have also been observed in the rhizosphere soils of other plants, such as *Ageratina adenophora* [50], *Broussonetia papyrifera*, *Ligustrum lucidum* [51]*, Artemisia ordosica* and *Caragana intermedia* [52]. Therefore, at the phylum level, they are more likely to constitute the predominant bacterial community in the majority of plant rhizosphere soils. Nevertheless, significant variations were observed in the relative abundances of dominant bacterial groups within the rhizosphere of *B. striata* across different regions. Notably, Actinobacteria exhibited the highest relative abundance in the *B. striata* rhizosphere of Lishui, Zhejiang. Furthermore, substantial difference were also noted in the enrichment of microbial groups at the genus level within the *B. striata* rhizosphere. For instance, Kaitobacter emerged as the dominant bacterial genus in Wuhu, Anhui and Jinzhai; meanwhile, Humicola was identified as the dominant fungal genus in Liupanshui, Guizhou, and Paraboeremia held dominance in Lishui, Zhejiang. It is noteworthy that Kaitobacter is a functional microbial group involved in carbon assimilation, and plays a pivotal role in ferrous oxidation-coupled carbon fixation processes. This not only contributes to an increase in organic carbon content within soil but also effectively mitigates heavy metal pollution risks [53]. Humicola can produce metabolites with unique biological activity and diverse structures [54], which may have an impact on the growth and development of *B. striata*. Paraboeremia is the most common and dominant fungal genus associated with the roots of Calanthe orchid species [55]. It is capable of symbiosis with orchids and plays a crucial role in their growth and development [56]. Furthermore, β diversity analysis showed that there were significant differences in the rhizosphere microbiota of *B. striata* from different habitats, indicating that geographical environment exerts a discernible influence on the composition of the rhizosphere microbiota.

The core microbiota, as a key component of the basic functions of the host holographic body, can not only directly play a beneficial role but can also influence a wider range of microbial communities through community cascade effects, thereby promoting the evolution and function of microbial communities [38, 57, 58]. We obtained five core rhizosphere microbial groups, Paraburkholderia, Methylibium, Bradyrhizobium, Chitinophaga, and Mycobacterium, through network coexistence analysis in the rhizosphere soil of eight different production areas. According to reports, Paraberkholderia demonstrates extensive metabolic capacity, with certain species possessing nitrogen fixation ability [59–61] and antifungal properties [62], particularly the potential for degradation of aromatic compounds, making it particularly suitable for bioremediation applications involving such compounds [63]. As a methylotrophic bacterium, Methylobacterium not only utilizes single carbon compounds as a carbon and energy source for growth but also metabolizes and produces various beneficial byproducts, such as plant hormones, iron carriers, and vitamin B12, as a result of promoting plant growth [64, 65]. Bradyrhizobium, the main nitrogen-fixing microbiota, can convert free nitrogen into nitrogen-containing compounds that can be directly absorbed and utilized by plants through biological nitrogen fixation, playing a significant role in plant growth and development. Research on the fruit bodies of ectomycorrhizal fungi found that Chitinophaga is almost a specific genus of bacteria that occurs almost exclusively in *Cantharelluss* and inferred that it is closely related to the functional requirements of *Cantharelluss* [66, 67]. Additionally, at the species level, we found that two of the five core microbes belonged to Paraburkholderia and two belonged to Bradyrhizobium. This suggests that species from the same genus tend to co-occur in similar ecological modules with comparable functions. Additionally, a majority of nodes in the rhizosphere microbial network exhibit positive correlations, indicating extensive coexistence and reciprocity among core microbes. These interactions collectively influence the growth and development of *B. striata*.

Cell chemotaxis, flagellar assembly, biofilm formation, and bacterial movement in the rhizosphere microbiome reflect the attraction of root exudates to microbes [68]. Interestingly, functional pathways related to flagella assembly, bacterial chemotaxis, and carbon fixation pathways of prokaryotes are significantly enriched within the rhizosphere microbiota associated with *B. striata*. As an ecological advantage strategy, bacterial chemotaxis indicates that bacteria are more likely to exhibit movement towards beneficial chemical gradients. Consequently, the rhizosphere secretions of *B. striata* may play a role in promoting the aggregation of specific microbes, thereby influencing the composition of rhizosphere microbiota. Furthermore, the functional pathways of rhizosphere microbes in *B. striata* may elicit heightened levels of ROS in the root, serving as a defense mechanism against potential pathogen invasion [69]. Previous studies have shown that glutamic acid can reshape the plant microbiota to enhance plant resistance against pathogens [70], and it is noteworthy that rhizosphere microbes exhibit abundant glutamate anabolism pathways. This suggests another potentially effective means by which rhizosphere microbes protect plants from pathogens. This “rhizosphere effect” is an important factor in shaping the rhizosphere microbiome, while rhizosphere microbes obtain nutrients through root exudates and derivatives.

Militarine is the sole indicator component of *B. striata* in the “Pharmacopoeia of the People’s Republic of China (2020)”, while *B. striata* polysaccharides serve as its primary functional components. Therefore, promoting the biosynthesis of militarine and BSP through rhizosphere microbial assembly holds significant practical production significance. Based on the functional characteristics of rhizosphere microbes from eight production areas, it was found that rhizosphere microbes in Jinzhai, Anhui and Liupanshui, Guizhou showed significant potential to promote the biosynthesis of militarine, whereas those in Guilin, Guangxi and Pan'an, Zhejiang exhibited notable potential to enhance BSP biosynthesis. Currently, based on the source species of gene annotation, it can be inferred that candidate microbes are involved in regulating militarine and BSP. However, further functional validation is needed to clarify the specific microbes involved and their contribution to component synthesis. Moreover, two of the candidate microbes related to militarine biosynthesis are identified as belonging to Burkholderia, suggesting their potential importance in promoting militarine bioaccumulation, which warrants further investigation. In addition, differences in the assembly of rhizosphere microbes and the abundance of functional genes may be critical factors influencing the quality variations of *B. striata*. Based on the results from all production areas, it was found that the levels of militarine and BSP in *B. striata* from Liupanshui, Guizhou, were significantly higher than those in other production areas, indicating higher quality. Meanwhile, *B. striata* from Liupanshui, Guizhou, showed significant potential for promoting militarine biosynthesis in rhizosphere microbes. Thus, we speculate that this may be attributed to the key gating effect of the root plane as stated by Edward et al. [71], that is, rhizosphere microbes enter plant roots and form endophytic microbes with selectivity, which in turn affects the accumulation of metabolites in the plant. In addition, the organic carbon and available nitrogen contents of soil in Liupanshui, Guizhou, were significantly higher than those of soil in other producing areas, providing an additional source of nutrition for the accumulation of BSP in *B. striata*.

Numerous studies have shown that soil microbial diversity and community structure are influenced by soil factors [72, 73]. The results of this study indicate that soil physical and chemical factors drive the composition of the rhizosphere microbiota in *B. striata*, with soil alkaline nitrogen and soil organic carbon being consequential factors affecting the microbiota. Pu Yang et al. [74] found that soil alkaline nitrogen is the strongest predictor of bacterial and fungal community composition, which is consistent with the results of this experiment. Previous studies have also highlighted the significant influence of soil factors particularly pH, on rhizosphere bacterial communities [75], aligning with the results of our structural equation model analysis. Soil organic carbon, available potassium, and pH indirectly affect the accumulation of *B. striata* metabolites by significantly affecting bacterial composition. Correlation analysis revealed a strong positive relationship between soil organic carbon and the contents of metabolites militarine, BSP, batatasin III, blestriarene A, and coelonin. With the exception of militarine and BSP, the remaining three metabolites were stilbene compounds. Accordingly, we speculated that soil organic carbon plays a vital role in promoting the accumulation of stilbene compounds. The stilbene compounds in plants not only have extensive biological activity [76] but also contribute to enhancing the plant's resistance to environmental stress [77], thereby playing a pivotal role in plant growth and development. Consequently, increasing the accumulation of stilbene compounds in *B. striata* by regulating the soil physicochemical properties holds significant practical implications for actual production. In addition, results from structural equation modeling also indicate that, compared to the composition of fungal communities, the composition of bacterial communities plays a more important role in the accumulation of metabolites. In fact, the absolute abundance of bacterial communities in rhizosphere microbial communities is much higher than that of fungal communities, and there exists a relationship between community function and abundance. Moreover, numerous studies have shown that rhizosphere bacteria and their metabolites not only impact plant growth and stress resistance, but also influence the synthesis of plant biological metabolites [78, 79]. For example, *Piriformospora indica* and *Azotobacter chroococcum* have been shown to increase the artemisinin content of *Artemisia caruifolia* [80]. In the present study, we observed a significant positive correlation between the function of rhizosphere microbes and the main functional components, such as BSP and militarine, in *B. striata*. Combined with gene analysis related to militarine and BSP biosynthesis, we concluded that the additional metabolic capacity provided by rhizosphere microbes and their genes related to the synthesis of medicinally active ingredients may contribute to additional metabolic capacity for *B. striata*. Overall, the results indicated that the metabolites of *B. striata* are jointly regulated by multiple factors, and there are certain mutual influences and indirect effects within these factors, ultimately acting together on the accumulation of metabolites.

## Conclusion

This study utilized metagenomic technology and targeted metabolomics technology to uncover the comprehensive regulation of rhizosphere microorganisms and secondary metabolites of *B. Striata* for the first time. To our knowledge, this is a novel report on the joint analysis of microbes and metabolites in the rhizosphere of *B. striata*, but the core and functional species have not yet been isolated from the rhizosphere soil, resulting in limited practical applications. Therefore, future research should focus on large-scale isolation and identification of relevant microorganisms, as well as validation to facilitate their development and application. In summary, identifying microbe-soil-metabolite interactions can help us select beneficial growth-promoting bacteria as biological fertilizers, enabling effective fertilization management to guide cultivation practices and improve the quality of *B. striata* while laying a foundation for future conservation research and agricultural sustainability.

## Methods

### Sample collection

In this study, eight geographic areas known for *B. striata* production were selected as sampling points across four provinces: Zhejiang Province, Anhui Province, Guangxi Province, and Guizhou Province. Details of the sampling points can be found in Table 2, with the sampling conducted in September 2021. All plant samples have been taxonomically as the orchid plant *Bletilla striata* (Thunb.) Reichb. f. by Professor Shuili Zhang from Zhejiang Chinese Medical University. Our team has obtained official permission for the collection of plant materials, and the voucher number (20211011-ND) and specimens are stored in the laboratory of the Institute of Traditional Chinese Medicine Resources at Zhejiang Chinese Medical University, maintained at a temperature of -80 ℃. Five healthy three-year-old *B. striata* plants were randomly selected from each sampling site. The humus was removed from the soil surface, and the roots of healthy plants were excavated vertically from 0 to 20 cm along the base of the plants. The soil around the roots was carefully shaken off, and then the roots were immersed in a sterile bottle containing sterilized phosphate buffer saline (pH = 7.4). They were continuously shaken to merge with the washing solution, forming a rhizosphere soil suspension. After centrifugation, the rhizosphere soil samples were stored at − 80 °C in an ultra-low temperature refrigerator for amplification and metagenomic detection. The fresh *B. striata* tubers were collected, cleansed through three rounds of ultrasonic cleaning with sterile water, and then stored at − 80 °C.

**Table 2:** List of sampling locations

Sample ID	Sampling location	Latitude (°N)	Longitude (°E)
AHJ	Jinzhai County, Anhui Province	31°43′38″	115°56′03″
AHW	Wuhu City, Anhui Province	31°20′27″	120°27′01″
ZLS	Lishui City, Zhejiang Province	28°26′45″	119°54′46″
ZJP	Pan'an County, Zhejiang Province	29°03′15″	120°27′01″
GXG	Guilin City, Guangxi Province	25°18′50″	110°18′07″
GXY	Guangxi Medicinal Botanical Garden	22°51′15″	108°22′06″
GZL	Liupanshui City, Guizhou Province	26°12′05″	105°28′49″
GZQ	Qiandongnan Miao and Dong Autonomous Prefecture, Guizhou Province	26°35′01″	107°59′03″

### Soil physicochemical parameters

The pH value was measured using the water extraction (soil water ratio of 2.5:1)—potential method; soil organic carbon content was determined using the potassium dichromate oxidation capacity method; alkali-hydrolyzable nitrogen (AN) content was assessed using the alkaline hydrolysis diffusion method; available phosphorus (AP) content was measured using the ICP-OES method; available potassium (AK) content was determined using the flame photometric method. The physical and chemical indicators of the soil samples from each production area were measured five times.

### DNA extraction, DNA sequencing, and metagenomic processing

Genomic DNA was extracted from the samples using the CTAB method, and its concentration and purity were assessed on a 1% agarose gel. The DNA was then diluted to a concentration of 1 ng/µL with sterile water. Subsequently, 16S rRNA genes of distinct regions (16S V3-V4) were amplified using the specific primers 341F (5’-CCTAYGGGRBGCASCAG-3’) and 806R (5’-GGACTACNNGGGTATCTAAT-3’) with the barcode. Additionally, the ITS1-1F region of the ITS rRNA gene was amplified using the universal primers ITS1-1F-F (CTTGGTCATTTAGAGGAAGTAA) and ITS1-1F-R (GCTGCGTTCTTCATCGATGC). The degree of DNA degradation, potential contamination, and DNA concentration were measured using an Agilent 5400 instrument (Agilent Technologies Co., Ltd., USA). Library construction and sequencing were completed by Wekemo Tech Group Co., Ltd. (Shenzhen, China).

### Microbiome data analysis

Raw data of bacteria, fungi, and viruses in the rhizosphere of *B. striata* were obtained by metagenomic sequencing using the Illumina Novaseq high-throughput sequencing platform. To ensure data reliability, raw sequencing data underwent preprocessing using Kneaddata software. Kraken2 and a self-built microbial database (sequences belonging to bacteria, fungi, archaea, and viruses were screened from the NT nucleic acid database and RefSeq whole-genome database of NCBI) were used to identify the species contained in the samples, and Bracken was used to predict the actual relative abundance of species in the samples. Kraken2 is the latest comparison software based on K-mer with 16,799 known bacterial genomes [81–84]. After undergoing quality control and de-hosting, the clean reads were aligned to the Uniref90 database using Humann2 software (based on Diamond). Annotation information and relative abundance tables from each functional database were obtained according to the corresponding relationship between Uniref90 ID and each database [85–88]. Subsequent analyses including abundance clustering, principal coordinate analysis (PCoA), and NMDS dimensionality reduction were conducted using online cloud platforms (https://www.bioincloud.tech).

### Determination of metabolic components in* B. striata* tubers

The *B. striata* tuber samples were cut, homogenized, passed through an 80 mesh sieve, and then stored at − 80 °C before being freeze-dried for later use. Subsequently, 0.200 g of freeze-dried powder was weighed and mixed with 10 mL of 50% ethanol, followed by incubation at 25 °C for 30 min. The extraction process involved ultrasound treatment at 40 °C (250 W, 60 kHz) for 30 min and subsequent centrifugation at 13,523 × g for 15 min. The supernatant was collected to determine the content of small-molecule compounds, while the precipitate was used for polysaccharide extraction and determination.

Briefly, 1 mL of the supernatant was absorbed and diluted to 2 mL with 50% ethanol to produce a sample solution with a mass concentration of 10 mg/mL dried tuber powder. The samples were passed through a 0.22-μM filter membrane for high performance liquid chromatography (HPLC) analysis. The contents of militarine (58,139–23-4, Chengdu Must Bio-Technology Co., Ltd., Chengdu, China), coelonin (82,344–82-9, Beijing Gersion Bio-Technology Co., Ltd, Beijing, China), batatasin III (56,684–87-8, Chengdu Must Bio-Technology Co., Ltd, Chengdu, China), blestriarene A (126,721–53-7, Chengdu Must Bio-Technology Co., Ltd., Chengdu, China), dactylorhin A (256,459–34-4, Chengdu Must Bio-Technology Co., Ltd., Chengdu, China), and gymnoside III (899,430–03-6, Shanghai Yuanye Bio-Technology Co., Ltd., Shanghai, China) in *B. striata* tubers from different regions were determined via HPLC. Chromatographic conditions: The analysis was performed using a Waters ACQUITYUPLC BEHC18 column (2.1 mm × 100 mm, 1.7 μm) with a mobile phase consisting of acetonitrile (A) and 0.1% formic acid water (B) for gradient elution. The gradient program was as follows: 0–5 min, 10% A; 5–10 min, 10–18.9% A, 10–30 min, 18.9–32% A; 30–70 min, 32–52% A; 70–72 min, 52% A; 72–73 min, 52–95% A; 73–74 min, 95–10% A; 74–80 min, 10% A. Detection was carried out at a wavelength of 270 nm with a flow rate of 1.0 mL/min and the column temperature maintained at 30 °C. The injection volume was set to be 10 µL.

The collected precipitate was suspended in 20 mL of water and extracted in a 90 °C water bath for 2 h to determine the polysaccharide content. An equal volume of anhydrous ethanol was then added to precipitate the polysaccharides. The resulting precipitate was centrifuged at 4 °C and 8000 rpm for 5 min, followed by removal of the supernatant. The remaining precipitate was quantitatively dissolved in water as the sample to be measured. Glucose was used as a standard, and the sulfuric acid-phenol method was used for color development. The absorbance values of each sample at a wavelength of 490 nm were measured using a UV spectrophotometer (EnSpire, PerkinElmer, USA), and the content of polysaccharides was calculated.

### Statistical analyses

The Chao1, Shannon, and Simpson indices were used to evaluate the α diversity of the microbial community in the rhizosphere. Differences were tested using one-way ANOVA, with multiple comparisons were performed using Bonferroni corrected *p* < 0.05. The Bray Curtis distance matrix was analyzed using similarity analysis (ANOSIM) in QIIME, with *n* = 999 permutations, and significance set at *p* < 0.05. PCoA was also performed based on the Bray Curtis dissimilarity matrix to visualize β diversity between groups in the rhizosphere microbiome. A microbial community interaction network was established to explore the interactions among rhizosphere microbes, focusing on microbial groups with a relative abundance greater than 0.01% and present in more than 75% of the samples across groups. In this coexisting network, a SparCC correlation coefficient *r* > 0.6 between two nodes indicated significant correlation between them. Redundancy analysis (RDA) was conducted to determine the environmental parameters associated with the structure of the rhizosphere microbiota. A structural equation model was established to analyze the effects of soil physicochemical properties and microbial communities on the metabolic products of *B. striata*. Graphical visualization was performed in R software using the "lavaan" and "pieceSEM" R packages [89].

### Supplementary Information

Supplementary Material 1. 

## Data Availability

The datasets generated and/or analysed during the current study are available in the NCBI repository [PRJNA1029928].
