# Supplementary material for: The rhizosphere microbiome and its influence on the accumulation of metabolites in Bletilla striata (Thunb.) Reichb. f
Source: BMC Plant Biol. 2024 May 17;24:409. doi: 10.1186/s12870-024-05134-0 (PMC11100225; doi:10.1186/s12870-024-05134-0)
Supplement: Supplementary file 1 — Supplementary Material 1. [file 12870_2024_5134_MOESM1_ESM.docx]

**Supplementary Figures**

**
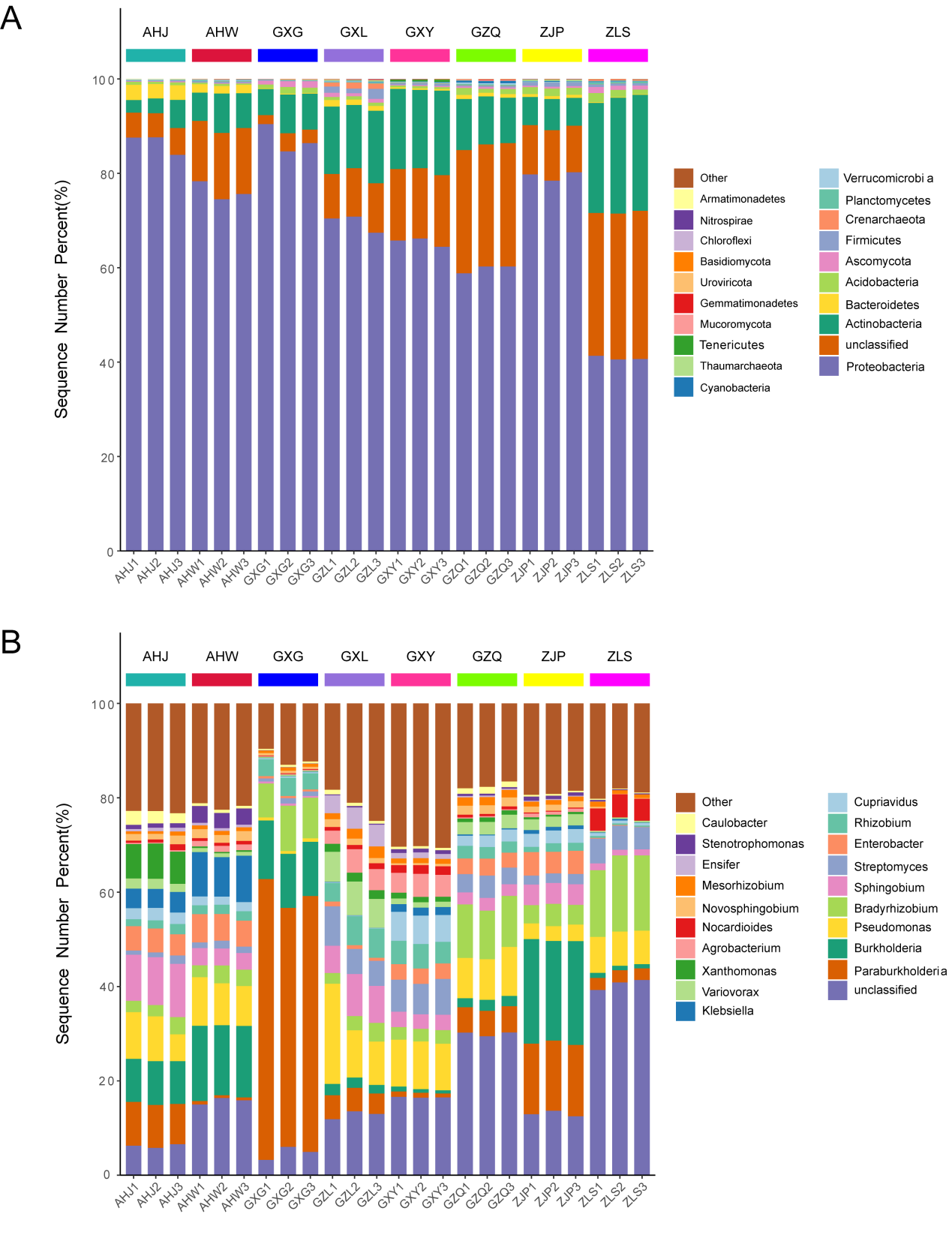
**

**Supplementary Figure 1.** Comparative analysis of the composition and diversity of rhizosphere microbial communities in *B. striata* from various geographical regions. (A) Relative abundance of rhizosphere microorganisms at the phylum level, as determined by metagenomic data. (B) Relative abundance of rhizosphere microorganisms at the genus level, as determined by metagenomic data.


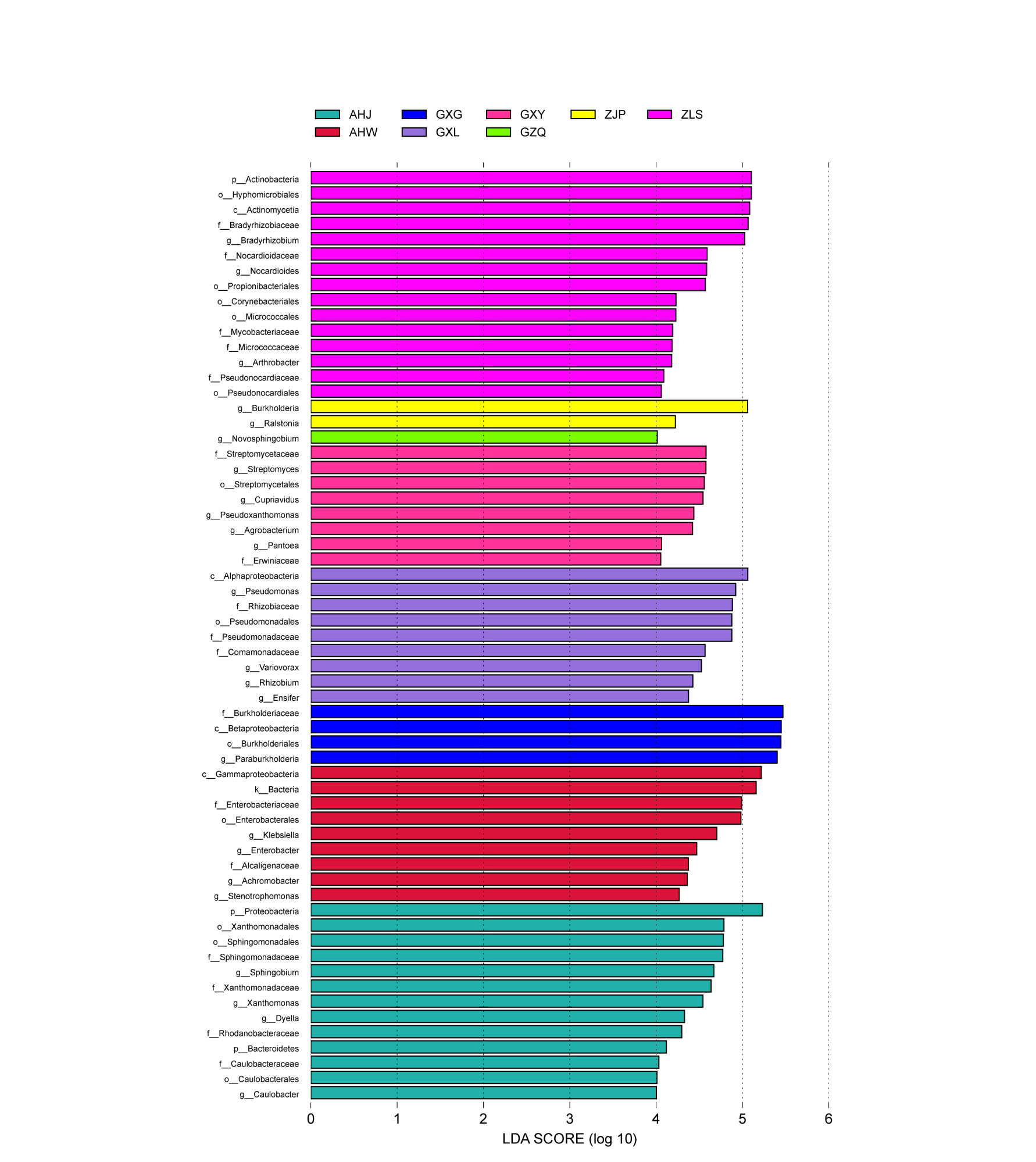


**Supplementary Figure 2.** LDA column chart of LEfSe analysis (LDA score > 4).

**
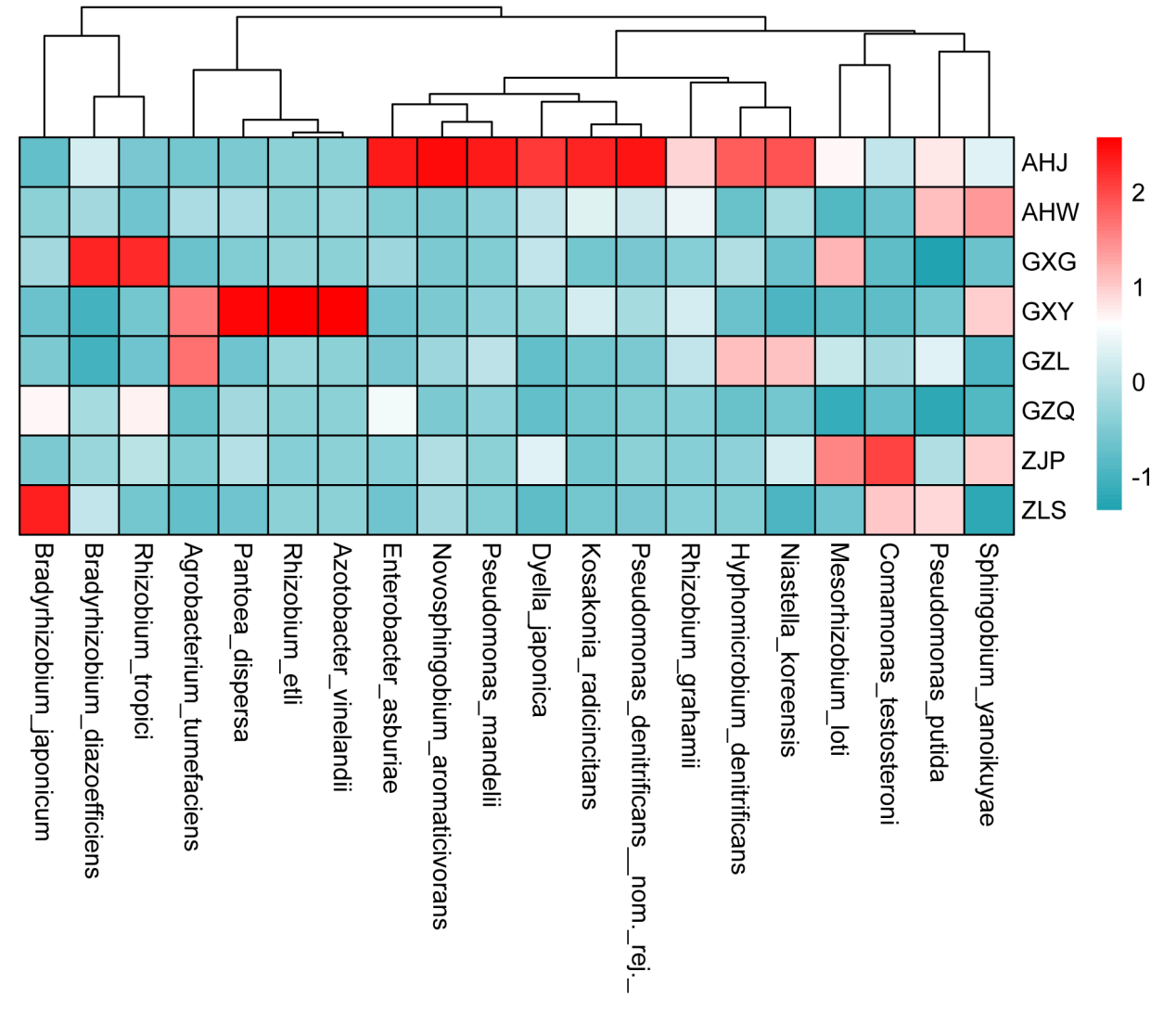
**

**Supplementary Figure 3.** Abundance heat map analysis of GAE gene annotated microorganisms in different regions.
